# Supplementary material for: Methodology to standardize heterogeneous statistical data presentations for combining time-to-event oncologic outcomes
Source: PLoS One. 2022 Feb 24;17(2):e0263661. doi: 10.1371/journal.pone.0263661 (PMC8870464; doi:10.1371/journal.pone.0263661)
Supplement: S1 Appendix — (DOCX) [file pone.0263661.s001.docx]

# Supplemental Appendix S1: Guyot SAS and R-code and examples

**SAS Code:**

/************************************************************************************/

/*The Guyot macro reconstruct the independent patient data (IPD) using */

/*digitized Kaplan-Meier curves with information about number of subjects */

/*at risk. */

/* */

/*The Guyot macro utilize %ARRAY and %DO_OVER written by Ted Clay and David Katz */

/*macros to iteratively calculate number of censored subjects and number */

/*events. More information about %ARRAY and %DO_OVER macros can be found at */

/*https://support.sas.com/resources/papers/proceedings/proceedings/sugi31/040-31.pdf*/

/* */

/*Please download and compile the macros from */

/*https://sasnrd.com/wp-content/uploads/2020/05/macros.txt before running the */

/*Guyot macros */

/* */

/************************************************************************************/

%include "User specified path...\ARRAY.sas";

%include "User specified path...\DO_OVER.sas";

%include "User specified path...\NUMLIST.sas";

**%MACRO** Guyot(

Digit, /*Specified the data that contains digitized x and y coordinates of Kaplan Meier curve*/

Nrisk, /*Specified the data that contains number of subjects at risk, r, and it's corresponding time, t*/

Digit_x, /*Column name of x coordinates in Digit*/

Digit_y, /*Column name of y coordinates in Digit*/

Nrisk_t, /*Column name of time, t in Nrisk*/

Nrisk_r, /*Column name of number of subjects at risk, r in Nrisk*/

trt /*Assign a name for this treatment*/);

/*Rename input data*/

data km_points(rename=(&Digit_x.=x &Digit_y.=y));

set &Digit.;

row+**1**;

run;

data time_at_risk(rename=(&Nrisk_t.=t &Nrisk_r.=r));

set &Nrisk.;

interval+**1**;

run;

/*Data preparation*/

proc sql noprint;

select max(x)

into :max_digit_x

from km_points;

/*Remove number at risk information beyond max(x)*/

data time_at_risk2;

set time_at_risk;

if t > &max_digit_x. then delete;

run;

data _NULL_;

set time_at_risk2 nobs=nint;

call symput("m_t"||put(left(interval), $3.), t);

call symput("nrisk"||put(left(interval), $3.), r);

call symputx('nint',nint);

run;

/* Create upper bound and lower bound for each interval time at risk*/

proc datasets library=work noprint;

delete lower upper temp temp2;

run;

%do i = **1** %to &nint.;

data temp;

set km_points;

if x >= &&m_t&i.;

t = &&m_t&i.;

run;

data temp(rename=(row=lower));

set temp nobs=nobs;

label row="lower";

if _n_=**1**;

run;

%if %sysfunc(exist(lower)) %then %do;

data lower;

set lower temp;

run;

%end;

%else %do;

data lower;

set temp;

run;

%end;

%end;

%do i = **1** %to &nint.;

data temp2;

set km_points;

if x < &&m_t&i.;

t = &&m_t&i.;

run;

data temp2(rename=(row=upper));

set temp2 nobs=nobs;

label row="upper";

if _n_=nobs;

run;

%if %sysfunc(exist(upper)) %then %do;

data upper;

set upper temp2;

run;

%end;

%else %do;

data upper;

set temp2;

run;

%end;

%end;

data lower;

set lower;

interval+**1**;

run;

data upper;

set upper;

interval+**1**;

run;

/* Attch lower and upper to time at risk*/

data time_at_risk3;

merge time_at_risk2 lower(keep = lower interval) upper(keep = upper interval);

by interval;

if upper = **.** then upper = lower;

run;

data time_at_risk4;

set time_at_risk3;

by interval;

set time_at_risk3(firstobs=**2** keep=r lower upper rename=(r=r_1 lower=lower_1 upper=upper_1))

time_at_risk3(obs=**1** drop=_all_);

label r_1 = 'r_1';

label lower_1 = 'lower_1';

label upper_1 = 'upper_1';

run;

proc sql;

create table time_at_risk5 as

select a.*, b.x as ts_lower label='ts_lower', b.y as s_lower label='s_lower', c.x as ts_lower_1 label='ts_lower_1', c.y as s_lower_1 label='s_lower_1'

from time_at_risk4 as a

left join km_points as b on a.lower = b.row

left join km_points as c on a.lower_1 = c.row;

quit;

proc sort data = time_at_risk5;

by interval;

run;

data km_points2;

set km_points;

n_hat = **.**;

cen = **0**;

d = **0**;

km_hat = **1**;

run;

/*First approximation of no. censored on interval i */

data time_at_risk5;

set time_at_risk5;

ncensor = round(r*s_lower_1/s_lower-r_1);

run;

/*Loop until Ncensor agrees with Number at risk*/

%LET RE_ADJ = 1;

%do %while (&RE_ADJ.>**0**);

data n_censor_guess(keep = count ncensor ts_lower ts_lower_1);

set time_at_risk5;

count+**1**;

if ncensor =**.** then delete;

run;

%***ARRAY***(N_CENSOR_GUESS, data = n_censor_guess, VAR=ncensor);

data cent;

%***DO_OVER***(N_CENSOR_GUESS, phrase = do i = **1** to ?; interval=?_i_; output; end;);

run;

%***ARRAY***(TS_LOWER, data = n_censor_guess, VAR=ts_lower);

%***ARRAY***(TS_NEXT, data = n_censor_guess, VAR=ts_lower_1);

data cent2;

set cent;

%***DO_OVER***(N_CENSOR_GUESS TS_LOWER TS_NEXT, phrase= if interval=?_i_ then cent= ?TS_LOWER+i*(?TS_NEXT-?TS_LOWER)/(?N_CENSOR_GUESS+**1**););

run;

proc sort data = km_points out=km_range; by descending x;run;

data km_range(keep = x_l x_u row);

set km_range;

x_l = x;

x_u = lag(x);

if x_u = **.** then delete;

run;

proc sort data = km_range; by row; run;

%***ARRAY***(X_L, data = km_range, VAR=x_l);

%***ARRAY***(X_U, data = km_range, VAR=x_u);

data cent3;

set cent2;

%***DO_OVER***(X_L X_U, phrase = if ?X_L <= cent < ?X_U then row = ?_i_;);

run;

proc sql;

create table cent_count as

select distinct row, count(row) as cen_count

from cent3

group by row;

proc sql;

create table km_points3 as

select * from km_points2;

update km_points3 as a

set cen=(select cen_count from cent_count as b

where a.row = b.row)

where a.row in (select row from cent_count);

proc sql;

create table km_points4 as

select * from km_points3;

update km_points4 as a

set n_hat=(select r from time_at_risk5 as b

where a.row = b.lower)

where a.row in (select lower from time_at_risk5);

data km_points5;

set km_points4;

retain km_hat2 **1** n_hat2;

if not missing(n_hat) then do;

d=round(n_hat*(**1**-(y/km_hat2)));

km_hat=km_hat2*(**1**-(d/n_hat));

n_hat2=n_hat-d-cen;

km_hat2=km_hat;

end;

else do;

n_hat = n_hat2;

d=round(n_hat*(**1**-(y/km_hat2)));

km_hat=km_hat2*(**1**-(d/n_hat));

n_hat2=n_hat-d-cen;

km_hat2=km_hat;

end;

run;

data km_points5;

set km_points5;

n_hat3 = lag(n_hat2);

if n_hat3 = **.** then n_hat3 = n_hat;

run;

%***ARRAY***(CENSOR_ADJ_ROW, data = time_at_risk4, var=lower);

data ncensor_adj(keep=row n_hat n_hat3 n_hat_diff);

set km_points5;

if row in (%***DO_OVER***(CENSOR_ADJ_ROW, phrase=?));

n_hat_diff = n_hat3 - n_hat;

run;

proc sql noprint;

select sum(abs(n_hat_diff))

into :RE_ADJ

from ncensor_adj;

proc sql;

create table time_at_risk5_1 as

select a.*, b.n_hat_diff

from time_at_risk5 as a left join ncensor_adj as b

on a.lower_1 = b.row

order by interval;

data time_at_risk5(drop = n_hat_diff);

set time_at_risk5_1;

ncensor = ncensor + n_hat_diff;

run;

%end;

/*No information to validate event and censor beyond last time at risk checkpoint*/

proc sql noprint;

select max(t)

into :last_check_point

from time_at_risk;

data final_result_trt&trt.(keep = x y row n_hat cen d trt);

set km_points5;

trt=&trt.;

if x > &last_check_point. and d > **1** then do; cen = d-**1**; d = **1**; end;

run;

/*Reconstruct IPD from finsal_result_trt*/

/*Eastimate censor_time*/

proc sort data = final_result_trt&trt.; by descending x; run;

data final_result_trt&trt.;

set final_result_trt&trt.;

lag_x = lag(x);

censor_time = (x+lag_x)/**2**;

run;

proc sort data = final_result_trt&trt.; by x; run;

data final_result_trt&trt.;

set final_result_trt&trt.;

retain _lag_x;

if not missing(lag_x) then _lag_x=lag_x;

else lag_x=_lag_x;

censor_time = (x+lag_x)/**2**;

drop _lag_x;

run;

data ipd_censor(keep= cen censor_time);

set final_result_trt&trt.;

if cen > **0**;

run;

proc sql noprint;

select min(n_hat), max(x)

into :censor_at_end1, :end_of_study_time1

from final_result_trt&trt.;

%***ARRAY***(CEN_EVENT, data = ipd_censor, VAR=cen);

%***ARRAY***(CENSOR_TIME, data = ipd_censor, VAR=censor_time);

data ipd_censor_trt&trt.(drop = i);

%***DO_OVER***(CEN_EVENT CENSOR_TIME, phrase=do i = **1** to ?CEN_EVENT; event=**0**; time2event = ?CENSOR_TIME; trt=&trt.; output;end;);

run;

data ipd_censor_at_end_trt&trt.(drop = i);

do i = **1** to &censor_at_end1.; event=**0**; time2event = &end_of_study_time1.; trt=&trt.; output; end;

run;

data ipd_event(keep = lag_x d);

set final_result_trt&trt.;

if d > **0**;

run;

%***ARRAY***(EVE_EVENT, data = ipd_event, VAR=d);

%***ARRAY***(EVE_TIME, data = ipd_event, VAR=lag_x);

data ipd_event_trt&trt.(drop = i);

%***DO_OVER***(EVE_EVENT EVE_TIME, phrase=do i = **1** to ?EVE_EVENT; event=**1**; time2event = ?EVE_TIME; trt=&trt.; output;end;);

run;

data ipd_trt&trt.;

set ipd_censor_trt&trt. ipd_censor_at_end_trt&trt. ipd_event_trt&trt.;

run;

**%MEND**;

**R Macro:**

An R macro written by Witold Wiecek to perform the Guyot algorithm can be found here: <https://rdrr.io/github/certara/survivalnma/src/R/guyot.R>

**Example of implementation of R macro:**

rm()

#Run Guyot macro first

#https://rdrr.io/github/certara/survivalnma/src/R/guyot.R

#Robotic

Tm1 <- c(0.000, 0.118, 0.216, 0.321, 0.401, 0.482, 0.587, 0.692, 0.797, 0.902, 1.006, 1.111, 1.216, 1.321, 1.426, 1.530, 1.635, 1.731, 1.826, 1.912, 1.997, 2.102, 2.207, 2.307, 2.407, 2.512, 2.617, 2.721, 2.826, 2.931, 3.036, 3.126, 3.217, 3.322, 3.426, 3.531, 3.636, 3.741, 3.841, 3.941, 4.046, 4.151, 4.227, 4.277, 4.379, 4.484, 4.589, 4.694, 4.798, 4.903, 4.979)

Km1<-c(1.000, 0.976, 0.962, 0.946, 0.924, 0.901, 0.880, 0.859, 0.845, 0.827, 0.808, 0.794, 0.774, 0.757, 0.754, 0.747, 0.735, 0.722, 0.703, 0.685, 0.662, 0.660, 0.657, 0.649, 0.631, 0.617, 0.606, 0.597, 0.596, 0.586, 0.580, 0.567, 0.549, 0.541, 0.540, 0.532, 0.522, 0.511, 0.489, 0.467, 0.464, 0.464, 0.456, 0.441, 0.441, 0.441, 0.441, 0.441, 0.441, 0.441, 0.441)

RskTm1 <- c(0, 1, 2, 3, 4)

NRisk1 <- c(300, 195, 124, 83, 27)

ipd_curve1 <- guyot.method(x = Tm1, y = Km1, t = RskTm1, r = NRisk1)

#Open

Tm2<-c(0,0.039, 0.120, 0.211, 0.287, 0.359, 0.449, 0.530, 0.606, 0.692, 0.797, 0.902, 1.006, 1.102, 1.197, 1.302, 1.407, 1.511, 1.616, 1.721, 1.807, 1.893, 1.997, 2.097, 2.197, 2.283, 2.369, 2.474, 2.579, 2.659, 2.740, 2.845, 2.950, 3.055, 3.160, 3.264, 3.369, 3.474, 3.579, 3.684, 3.789, 3.889, 3.963, 4.051, 4.112, 4.189, 4.246, 4.351, 4.455, 4.560, 4.651, 4.713, 4.817, 4.894)

Km2<-cc(1,0.987, 0.968, 0.947, 0.920, 0.890, 0.870, 0.839, 0.817, 0.793, 0.774, 0.763, 0.755, 0.739, 0.716, 0.705, 0.692, 0.674, 0.661, 0.642, 0.622, 0.600, 0.589, 0.574, 0.559, 0.542, 0.520, 0.504, 0.492, 0.471, 0.454, 0.453, 0.447, 0.442, 0.421, 0.413, 0.404, 0.404, 0.400, 0.391, 0.382, 0.370, 0.351, 0.347, 0.322, 0.318, 0.293, 0.292, 0.292, 0.292, 0.288, 0.257, 0.256, 0.256)

RskTm2 <- c(0, 1, 2, 3, 4)

NRisk2 <- c(300, 178, 102, 50, 16)

ipd_curve2 <- guyot.method(x = Tm2, y = Km2, t = RskTm2, r = NRisk2)

library(survival)

ipd_curve1$patient$treatment <- "1 robot blue"

ipd_curve2$patient$treatment <- "2 open green"

ipd <- rbind(ipd_curve1$patient, ipd_curve2$patient)

fit <- survfit(Surv(time, event) ~ treatment, data = ipd)

fit

summary(fit)

#par(mfrow=c(2,2))

plot(fit, col = c("blue","green"),ylim=c(0,1),lwd=2,lty=c(1,1))

lrtest <- survdiff(Surv(time,event)~treatment, data = ipd) #log-rank test

pvalue <- round(1-pchisq(lrtest$chisq,length(fit$strata)-1),digits=3)

lrsum<-paste("Overall Log rank statistic = ", round(lrtest$chisq,digits=2),", p=",pvalue)

text(2,.9,paste("Log-rank stat. = ", round(lrtest$chisq,digits=1),", p=",pvalue),cex=.8)

mod1<-coxph(Surv(time,event)~treatment, data = ipd)

summary(mod1)
