# Supplementary material for: Methodology to standardize heterogeneous statistical data presentations for combining time-to-event oncologic outcomes
Source: PLoS One. 2022 Feb 24;17(2):e0263661. doi: 10.1371/journal.pone.0263661 (PMC8870464; doi:10.1371/journal.pone.0263661)
Supplement: S2 Appendix — (DOCX) [file pone.0263661.s002.docx]

# Supplemental Appendix S2: Validation of Guyot Algorithm

*Introduction:*

Hazard ratio (HR) is considered the most appropriate statistic for meta-analysis of time dependent outcomes, but may not be provided in published literature. We developed hierarchical decision tree containing 4 methods for obtaining HR. The Guyot algorithm[10], which is recommended by the Cochrane handbook to estimate HR, combines digitized data from published Kaplan-Meier (KM) curves with information about the number of subjects at risk (Nrisk) to reconstruct approximate individual patient data (IPD) for time-to-event outcomes. We evaluated whether the Guyot Algorithm produced sufficiently accurate estimates under a range of conditions.

*Methods:*

*Figure 1. Calculation of Hazard Ratio from Real and Reconstructed Individual Patient Data (Click to enlarge image)*


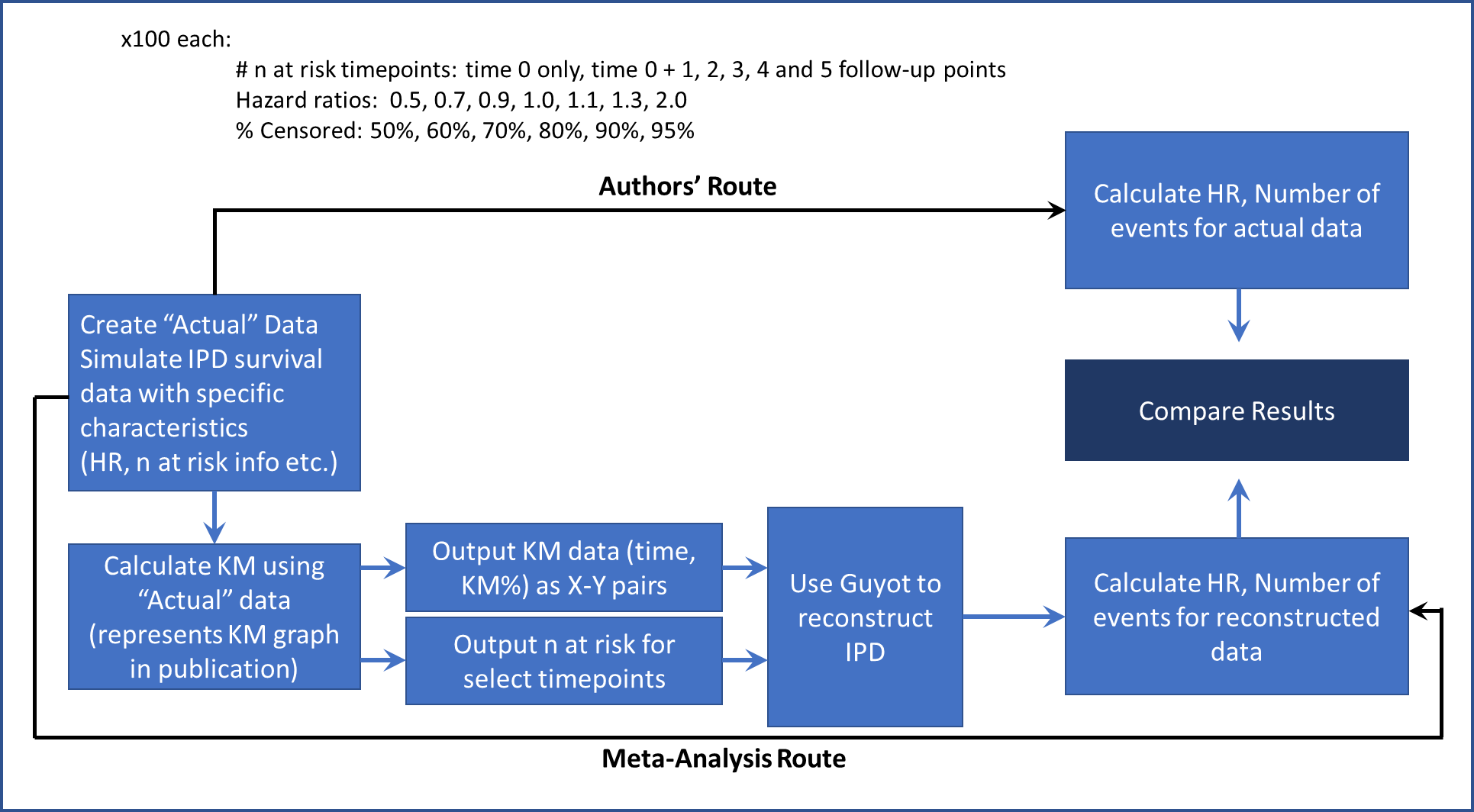


We simulated 1,900 time-to-event data sets with two treatment groups and 300 subjects in each group. To determine the impact on accuracy of Nrisk availability, the magnitude of the hazard ratio and the amount of censoring, these conditions were varied when producing the simulated data sets. Figure 1 shows the steps in the simulation and validation process.

HRs were varied from 0.5 to 2.0, censoring rates were varied from 50% to 95%, and the number of time points with Nrisk were varied from 0 to 5.

KM survival estimates and the Nrisk were exported from each simulated data set and used as input to the Guyot algorithm. The HR was calculated in both the simulated and the reconstructed IPD using Cox-Proportional Hazards. The difference between the simulated HR and reconstructed HR was used to quantify the accuracy of the Guyot algorithm and summarized as inter-quartile range (IQR) and by percentage change (PC, difference/real HR). The algorithm was executed and validated in R2 and SAS*.

* For SAS version of Guyot algorithm, please see Appendix S1.

*Results:*

*Figure 2: Difference in HR by Amount of N at Risk Information at Select Conditions (Click to enlarge image)*


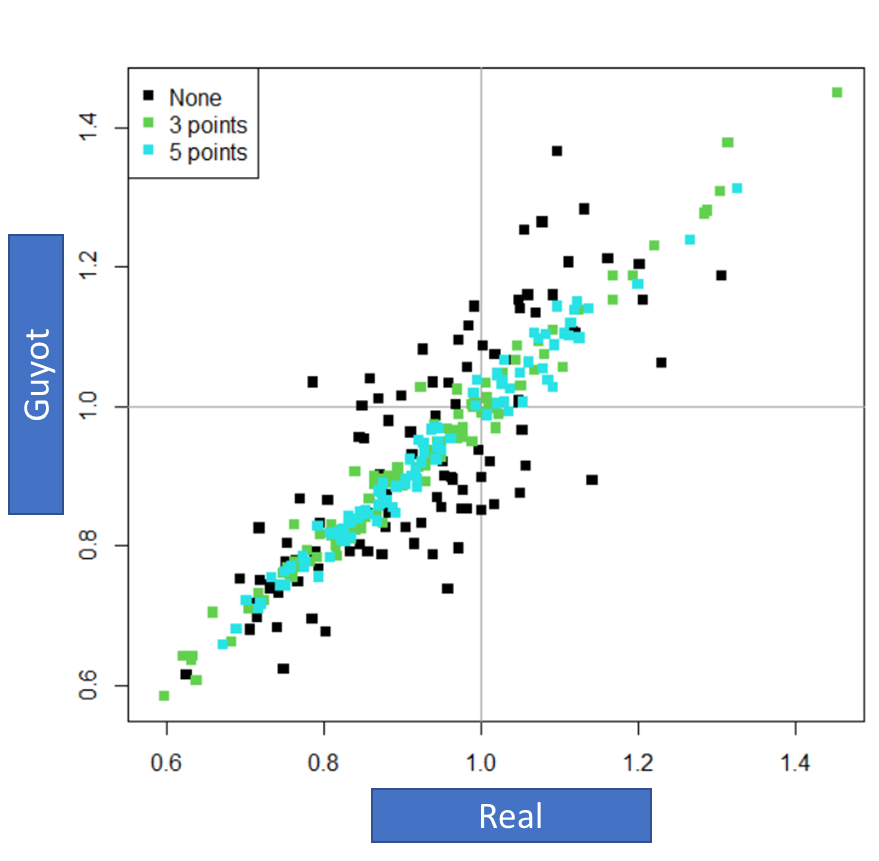


When Nrisk were varied from 0 to 5, the results show that as long as at least 1 follow-up time-point is available, the difference between real and reconstructed HR was IQR [-.03 to .03] and PC [median -.1%, IQR -1.7% to 1.6%]. When only time zero is available, the difference in HR in the simulations was IQR [-.07 to .07] and PC [median -1%, IQR -8% to 8%], but there were outliers that differed by as much as 0.27 (PC 24%). These cases can be identified and adjusted using the log-rank statistic.

*Figure 3: Difference in HR by Amount of Censoring at Select Conditions (Click to enlarge image)*


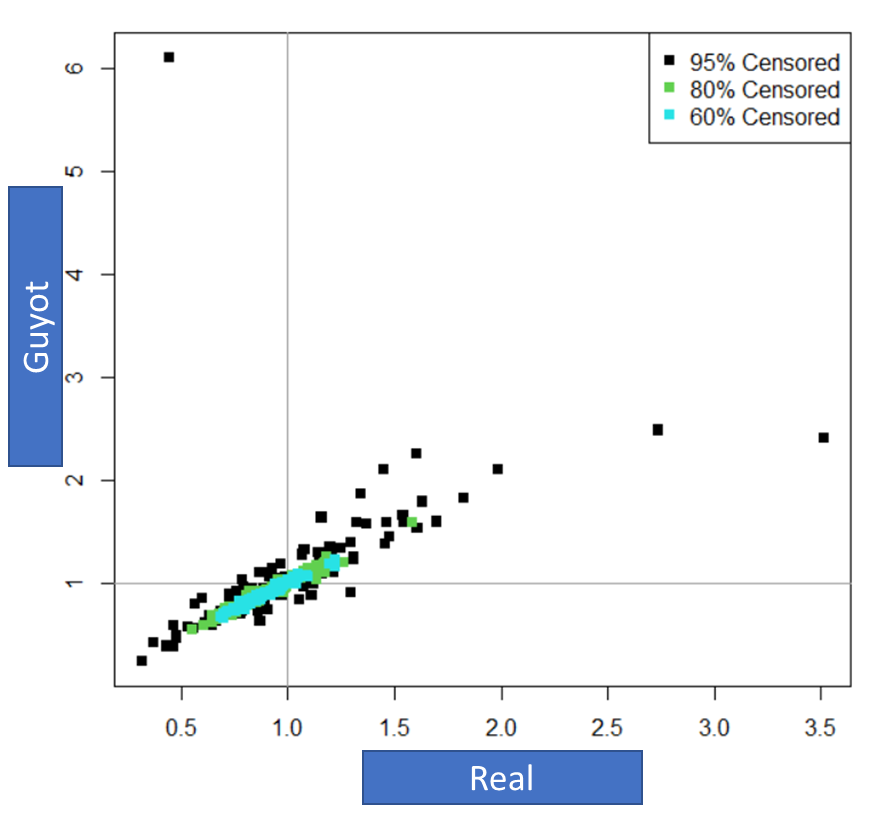


To assess the impact of the amount of censoring, data sets were simulated for the following levels of censoring: 50%, 60%, 70%, 80%, 90% and 95%. For data sets with up to 80% of censoring, the HR difference was IQR [-.02 to .02] and PC [median 0.5%, IQR -1.8% to 2.4%]. At 90% censoring, HR difference was IQR [-.05 to .04] and PC [median 0.9%, IQR -4.7% to 4.5%]. At 95% censoring, HR difference was IQR [-.05 to .13] and PC [median 3%, IQR -5% to 13.6%]. These cases can be identified and adjusted using the log-rank statistic.

*Figure 4: Difference in HR by Magnitude of Hazard Ratio at Select Conditions (Click to enlarge image)*


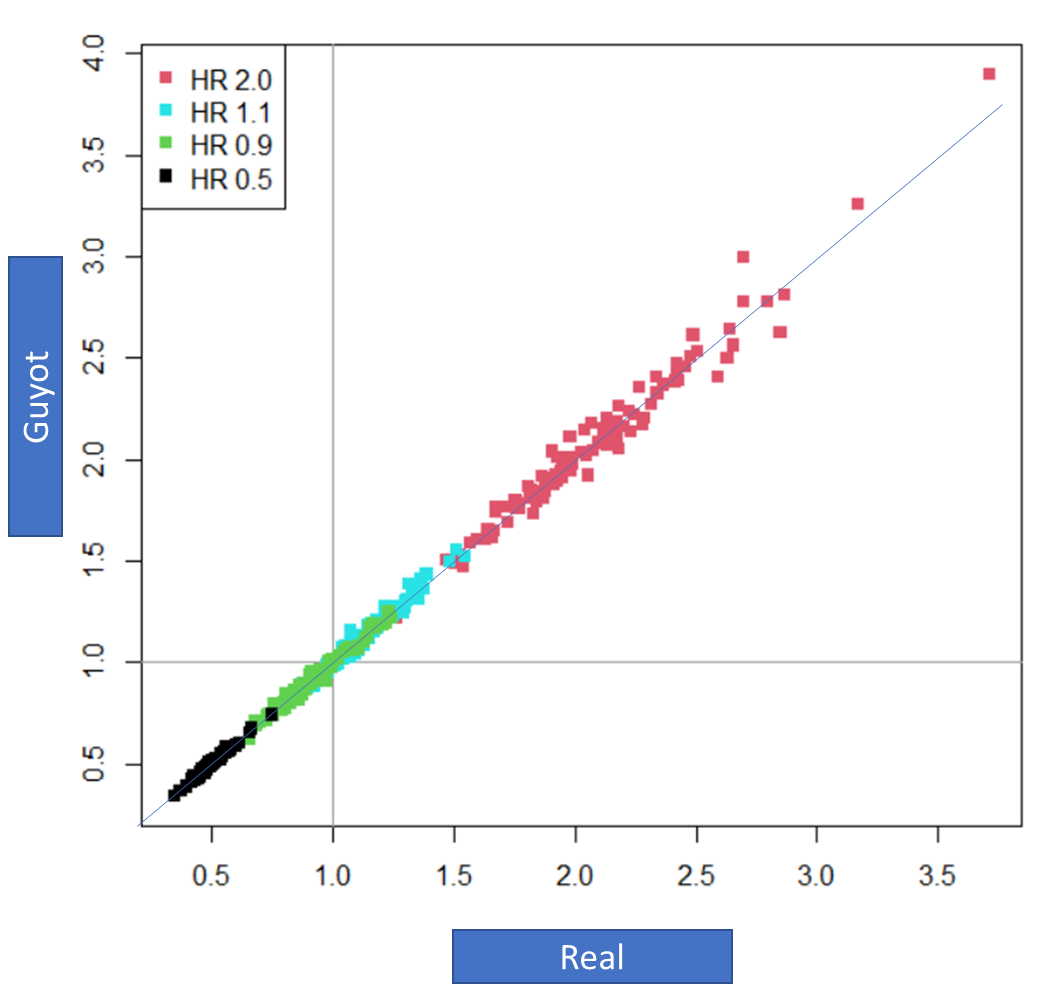


The accuracy was assessed using 700 simulated data sets with the following hazard ratios:  0.5, 0.7, 0.9, 1.0, 1.1, 1.3 and 2.0. These results showed that the difference in HR was IQR [-.04 to .03] and PC [median 0%, IQR -1.8% to 1.6%]. The difference is symmetric in that the hazard ratio is over- and under-estimated a similar proportion of the time, but the magnitude of the difference seems to increase in the hazard ratio.

*Summary & Recommendations:*

The Guyot algorithm can be implemented in SAS and R. We conducted the validation study to understand the performance of the algorithm and it’s limitations. We found that across censoring distributions, the difference between actual and reconstructed HR was IQR [-.05 to .13]. Across HRs, the IQR was -.04 to .03. When no information for Nrisk was given, the IQR of the difference was -.07 to .07. When ≥1 Nrisk time point available, the IQR of the difference was -.03 to .03. The Guyot algorithm is a viable method to estimate HR from KM curves, and it is preferable to the exclusion of the papers that did not present HR.

*Limitations & Conclusions:*

An important limitation is that this validation was performed using simulated data and sample sizes of 300 in each arm. Whether this algorithm works well in smaller sample size is not addressed by this analysis. Because the need for meta-analysis is the most pressing when there are several small studies that could potentially be combined, performance in small samples is an important consideration. Also, the KM time points could be extracted precisely from the simulated data so errors introduced in the digitization process were not considered.

Derivation of an estimate of the HR from KM curves was required for nearly a third of qualifying publications identified in the literature review. This exercise provides a validation of select aspects of the Guyot macro, which is an application of the recommended algorithm for this derivation. The analyses showed that the hazard ratios derived from reconstructed IPD using the Guyot algorithm are relatively consistent estimates of the real hazard ratio under most conditions observed in our analyses. The largest differences between the real and reconstructed hazard ratio occurred when no data were available for Nrisk, and where the amount of censoring exceeded 95%.

[1] Guyot P, Ades AE, Ouwens MJNM, Welton NJ. Enhanced secondary analysis of survival data: reconstructing the data from published Kaplan-Meier survival curves. BMC MED RES METHODOL. 2012;12(1):9-.
