# Supplementary material for: Methodology to standardize heterogeneous statistical data presentations for combining time-to-event oncologic outcomes
Source: PLoS One. 2022 Feb 24;17(2):e0263661. doi: 10.1371/journal.pone.0263661 (PMC8870464; doi:10.1371/journal.pone.0263661)
Supplement: S3 Appendix — (DOCX) [file pone.0263661.s003.docx]

# Supplemental Appendix S3: R functions for HR estimate calculations

**#Method 1: reverse reference group**

RevRef <- function(HR.Ref1, HRCIL.Ref1, HRCIU.Ref1) {

Hazard.Ratio <- c(HR.Ref1, 1/ HR.Ref1)

HRCIL.Ref2 <- c(HRCIL.Ref1, 1 / 1/HRCIU.Ref1)

HRCUL.Ref2 <- c(HRCIU.Ref1, 1 / 1/HRCIL.Ref1)

return(data.frame(reference = c("Group 1", "Group 2"),

Hazard.ratio = Hazard.Ratio, HR.lower = HRCIL.Ref2, HR.upper = HRCUL.Ref2))

}

#Example of function call

#Syntax: RevRef(HR original ref, HR CI lower (orig.), HR CI upper (orig.))

#Example:

RevRef(1.47, 1.14, 1.90)

##############

**#Method 1: Deriving CI from Reported HR and P-value**

GetCI <- function(HR, Pval, zScore = 1.96) {

SELnHR <- log(HR)/qnorm((1-Pval/2), mean=0, sd=1)

HR.CI1.TMP <- exp(log(HR) - SELnHR*zScore)

HR.CI2.TMP <- exp(log(HR) + SELnHR*zScore)

HR.CIL=min(HR.CI1.TMP,HR.CI2.TMP)

HR.CIU=max(HR.CI1.TMP,HR.CI2.TMP)

return(data.frame(Hazard.ratio = HR, HR.lower = HR.CIL,

HR.upper = HR.CIU, P.value = Pval))

}

#Example of function call

#Syntax: GetCI(Hazard Ratio, Log-Rank p-value)

#Example HR<1:

GetCI(.68, 0.0032)

#Example HR>1:

GetCI(1.47, 0.0032)

**#Method 2: Hazard Ratio as calculated using event counts**

M2EvCt <- function(Rr, Rc, Or, Oc, Pval, zScore = 1.96) {

Dth.r <- Or / Rr

Dth.c <- Oc / Rc

Dth.Diff = Dth.r - Dth.c

Pmult <- ifelse(Dth.Diff < 0, -1, 1)

inv.Vr <- 1/(((Or + Oc)*Rr*Rc)/((Rr + Rc)^2))

O_E <- (sqrt((Or + Oc)*Rr*Rc)/(Rr + Rc)*(qnorm((1-Pval/2), mean=0, sd=1))*Pmult)

LnHR <- O_E*inv.Vr

HR <- exp(LnHR)

HR.CIL <- exp (LnHR - zScore*sqrt(inv.Vr))

HR.CIU <- exp (LnHR + zScore*sqrt(inv.Vr))

return(data.frame(Hazard.ratio = HR, HR.lower = HR.CIL,

HR.upper = HR.CIU, P.value = Pval))

}

#Example of function call

#Syntax: M2EvCt(Total group 1 n , Total group 2 n, # events group 1, # events group 2, Log-Rank p-value)

#Example:

M2EvCt(300, 300, 105, 133, 0.003)

**#Method 4a: Hazard Ratio as calculated using KM estimates**

M4aHR.KM <- function(Rr, Rc, Surv.KMr, Surv.KMc, Pval, zScore = 1.96) {

Or <- (Rr - (Rr*Surv.KMr))

Oc <- (Rc - (Rc*Surv.KMc))

Dth.r <- Or / Rr

Dth.c <- Oc / Rc

Dth.Diff = Dth.r - Dth.c

Pmult <- ifelse(Dth.Diff < 0, -1, 1)

inv.Vr <- 1/(((Or + Oc)*Rr*Rc)/((Rr + Rc)^2))

O_E <- (sqrt((Or + Oc)*Rr*Rc)/(Rr + Rc)*(qnorm((1-Pval/2), mean=0, sd=1))*Pmult)

LnHR <- O_E*inv.Vr

HR <- exp(LnHR)

HR.CIL <- exp (LnHR - zScore*sqrt(inv.Vr))

HR.CIU <- exp (LnHR + zScore*sqrt(inv.Vr))

return(data.frame(Hazard.ratio = HR, HR.lower = HR.CIL,

HR.upper = HR.CIU, P.value = Pval))

}

#Example of function call

#Syntax: M4aHR.KM(Total group 1 n , Total group 2 n, KM Survival group 1, KM Survival group 2, Log-Rank p-value)

#Example:

M4aHR.KM(300, 300, 0.586, 0.445, 0.003)

##############

**#Method 4b. Hazard Ratio calculated using median survival estimates**

M4bMedSurv <- function(Or, Oc, MSr, MSc, zScore = 1.96) {

HR <- MSc/MSr

SELnHR <- sqrt((1/Or)+(1/Oc))

LnHR <- log(HR)

HR.CIL <- exp(LnHR - zScore*SELnHR)

HR.CIU <- exp(LnHR + zScore*SELnHR)

return(data.frame(Hazard.ratio = HR, HR.lower = HR.CIL,

HR.upper = HR.CIU))

}

#Example of a function call

#Syntax: M4bMedSurv(# events group 1, # events group 2, Median survival group 1, Median survival group 2)

#Example:

M4bMedSurv(105, 133, 3.8, 2.5)
