# Supplementary material for: Methodology to standardize heterogeneous statistical data presentations for combining time-to-event oncologic outcomes
Source: PLoS One. 2022 Feb 24;17(2):e0263661. doi: 10.1371/journal.pone.0263661 (PMC8870464; doi:10.1371/journal.pone.0263661)
Supplement: S4 Appendix — (DOCX) [file pone.0263661.s004.docx]

# Supplemental Appendix S4: Assumptions, Rules, and Tips

**Overall**

- Assumption: Hazard ratios are a valid comparison of overall risk between groups in directionality and magnitude even when the hazards are not proportional, but statements quantifying the comparisons (e.g., a 5 x higher risk) should not be made in that case.
  - Our Rule: All available data, outcome definitions, and stated conclusions were utilized to determine the most valid data, method, and p-value to use, and to check the accuracy of Method 2-4 calculations.
  - Our Rule: When there was a judgement call needed, we selected the method that was the most conservative (most disfavored) for the robotic surgical approach.

**Method 1**

- Assumption: The reference group for the hazard ratio for each study and outcome (each comparison) needs to be the same to pool the data.
  - Our Rule: Standardize the reference group to the non-robotic group.
    - Tip: to swap reference groups: 1/HR [1/CI high, 1/CI low].
    - Tip: to determine reference groups: see Table 1.
- Assumption: Determine criteria *a priori* for choosing a hazard ratio when more than one is provided (e.g., unmatched vs. matched).
  - Our Rule: Use an adjusted or matched HR over an unmatched HR. In instances where both an adjusted and a matched HR are provided, to maximize group size, use an adjusted HR using the whole patient population over a matched patient cohort when matching decreased the sample size. Analyses using entire populations account for the relative frequency of case types, severity of disease, surgeon experience, etc. and the results are more generalizable. For sensitivity analyses, use the hazard ratio from the largest sample that adequately addresses confounding.

**Method 2**

- Assumption: all reported p-values are two-tailed.
  - Our Rule: equation V should reflect the directionality of the difference.
    - Tip: multiply equation V by +1 if the robotic death rate is greater, and by -1 if the robotic death rate is less.
    - Tip: or modify equation V by using ${}^{-1}(1-\frac{p}{2})$ or ${}^{-1}(\frac{p}{2})$ to get a positive value when the robotic death rate is greater and negative when the robotic death rate is less.
- Assumption: Total sample size (N) is equal to “n at risk” at time zero unless otherwise stated.
  - - Tip: “n at risk” at time zero = N - # patients lost to follow-up.
- Assumption: Total number of dead can be calculated if not provided.
  - - Tip: back calculate from proportional death rate (N x rate=#dead).
    - Tip: add death due to disease (DOD) and death due to other (DOO).
    - Tip: subtract number of alive from the total sample size.
- Assumption: Disease-free survival can be calculated if not provided if there is no evidence it would be inaccurate.
  - - Tip: add DOO and DOD and alive with disease (AWD)
    - Tip: add overall mortality and (total recurrence minus DOD)

**Method 3**

- Assumption: The Kaplan-Meier (KM) curve shows complete follow-up and image quality is sufficient for digitization.
  - - Tip: if # events is few enough, may be able to manually count them.
    - Tip: If no KM curve is shown, but the time of each event was reported along with summary information about the follow-up distribution, an approximation to the KM curve can be constructed manually.
    - Tip: p-value can be used to adjust censoring in the Guyot algorithm when n at risk over time is not reported.

**Method 4**

- Kaplan-Meier Estimate Assumptions Required: That there was no censoring, that the survival curves do not cross after the estimated time point, and that the hazards are relatively proportional.
  - Our Rule: The conclusions of the authors were used to determine if this approach would accurately reflect the overall comparison between cohorts.
    - Tip: # dead ~ N-(N x Kaplan-Meier survival estimate)
- Assumptions: Determine *a priori* which timepoint will be used when several Kaplan-Meier survival estimates are provided (ie. 2-year, 3-year, and 5-year).
  - Our Rule: use the latest timepoint.
- Median Survival Assumptions: Using the median survival works best when the cohort sizes are similar, when there is a constant event rate, and is only available when there is enough follow-up and deaths to reach a 50% survival rate in all groups.

**p-value**

- Assumption: Kaplan-Meier log-rank p-value in conjunction with an HR, a KM curve, or a KM survival estimate, can be used to estimate the HR with the other Methods 2-4.
  - Our Rule: P-values where the statistical test used was not explicitly stated were assumed to be log-rank if an attempt to replicate the reported p-value using Chi2 or Fisher's exact tests failed.
    - Tip: When using a p-value to calculate an HR or CI, if it is reported as “less than” (e.g., p<0.001), check for a log-rank statistic and use if reported. If no log-rank statistic is reported, the convention is to treat it as equal (e.g., p<0.001 becomes p=0.001).
- Assumption: A 3-way (or more) p-value is testing the overall null hypothesis that there are no differences in survival curves. This may not accurately reflect individual pairwise comparisons.
  - Our Rule: In cases where an overall p-value (comparing more than two groups) is the only p-value given, it might be a reasonable approximation of the pairwise test when the curves look similar (are overlapping). If this assumption is not valid, calculate pairwise p-values using the Guyot reconstructed data from the KM curve.

**Biochemical Recurrence (BCR) in Prostate Cancer**

- Assumption: If an author calls the data: disease-free survival (DFS), recurrence-free survival (RFS), or biochemical recurrence-free survival (BCRFS), they included any deaths, unless otherwise stated.
- Assumption: All patients who died of disease have already been counted in the recurrence rate.
  - Our Rule: When both overall recurrence and biochemical recurrence were reported, use the decision tree to choose the highest level of data (ie. pull the hazard ratio for biochemical recurrence before using the event n for overall recurrence). If the level of data quality was equal, use overall recurrence.
- Assumption: When a paper reported BCR or BCRFS and did not mention any deaths, treat the lack of mention as a statement that there were no deaths, and use the data interchangeably for DFS and recurrence (using the highest method available for either), unless the paper is a large sample size database paper (because it is more likely to have unmentioned deaths).
