# Supplementary material for: Methodology to standardize heterogeneous statistical data presentations for combining time-to-event oncologic outcomes
Source: PLoS One. 2022 Feb 24;17(2):e0263661. doi: 10.1371/journal.pone.0263661 (PMC8870464; doi:10.1371/journal.pone.0263661)
Supplement: Attachment — Submitted filename: Response to Reviewers.docx [file pone.0263661.s006.docx]

Reviewers' comments:

Reviewer's Responses to Questions

**Comments to the Author**

1. If the authors have adequately addressed your comments raised in a previous round of review and you feel that this manuscript is now acceptable for publication, you may indicate that here to bypass the “Comments to the Author” section, enter your conflict of interest statement in the “Confidential to Editor” section, and submit your "Accept" recommendation.

Reviewer #3: (No Response)

2. Is the manuscript technically sound, and do the data support the conclusions?

The manuscript must describe a technically sound piece of scientific research with data that supports the conclusions. Experiments must have been conducted rigorously, with appropriate controls, replication, and sample sizes. The conclusions must be drawn appropriately based on the data presented.

Reviewer #3: Yes

3. Has the statistical analysis been performed appropriately and rigorously?

Reviewer #3: Yes

4. Have the authors made all data underlying the findings in their manuscript fully available?

The [PLOS Data policy](https://urldefense.com/v3/__https:/eur01.safelinks.protection.outlook.com/?url=http*3A*2F*2Fwww.plosone.org*2Fstatic*2Fpolicies.action*23sharing&data=04*7C01*7Capril.slee.15*40ucl.ac.uk*7C17102949ba8d4c59308d08d9bb34667e*7C1faf88fea9984c5b93c9210a11d9a5c2*7C0*7C0*7C637746656898867496*7CUnknown*7CTWFpbGZsb3d8eyJWIjoiMC4wLjAwMDAiLCJQIjoiV2luMzIiLCJBTiI6Ik1haWwiLCJXVCI6Mn0*3D*7C2000&sdata=FgXv5HKANh9XhXMvYR8hX*2Fzy*2FQOnuSIzkKWPfkBavAE*3D&reserved=0__;JSUlJSUlJSUlJSUlJSUlJSUlJSUl!!PEK3uBjM8x5RqC4b!VCUVNE2KXJkvHoN9Qf6qKNYaK5iw4_QkJEgmasCYh_xPZ5rN074C0HMgxAkXy338QDA$) requires authors to make all data underlying the findings described in their manuscript fully available without restriction, with rare exception (please refer to the Data Availability Statement in the manuscript PDF file). The data should be provided as part of the manuscript or its supporting information, or deposited to a public repository. For example, in addition to summary statistics, the data points behind means, medians and variance measures should be available. If there are restrictions on publicly sharing data—e.g. participant privacy or use of data from a third party—those must be specified.

Reviewer #3: No

5. Is the manuscript presented in an intelligible fashion and written in standard English?

PLOS ONE does not copyedit accepted manuscripts, so the language in submitted articles must be clear, correct, and unambiguous. Any typographical or grammatical errors should be corrected at revision, so please note any specific errors here.

Reviewer #3: Yes

6. Review Comments to the Author

Please use the space provided to explain your answers to the questions above. You may also include additional comments for the author, including concerns about dual publication, research ethics, or publication ethics. (Please upload your review as an attachment if it exceeds 20,000 characters)

**Reviewer #3:** As someone who's worked on extracting this kind of data for systematic reviews, I was happy to see this outline of different ways to extract survival outcomes from publications.

**Reviewer #3 Comment #1:** It was especially nice to see R code for the method of Guyot et al (in the supplement). The worked examples will also certainly be helpful for many researchers doing this sort of data extraction. Would it be possible to include R and / or SAS code for these examples in the supplement? This would further aid researchers in performing their data extraction without mistakes in simple bits of code. For example, I see that the function NORM.S.INV was used, but I'm not sure without looking it up in both excel and R help files which is the corresponding R function.

Response to Reviewer #3 Comment #1: We originally performed the HR estimate calculations in excel only; however, we went back and created code de novo in R for all of the equations needed for Methods 1-4, and we are providing this R code in the supplement as appendix S3 and re-naming the tips and tricks appendix as S4. This new appendix is cited in the “worked example” section of the paper as follows:

“We are also providing the R code in Supplemental Appendix S3.”

And in the discussion as follows:

“Our methods add to the list of previously published techniques for analyzing time-to-event outcomes (4, 7,18), and include details for implementing our strategies (Supplemental Appendices – S1 Guyot code, S3 R code for equations of interest, and S4 Data extraction & tricks).”

The addition to the supplement is as follows:

Supplemental Appendix S3: R functions for HR estimate calculations

**#Method 1: reverse reference group**

RevRef <- function(HR.Ref1, HRCIL.Ref1, HRCIU.Ref1) {

#Display for HR (CI) as entered

DISP1<-paste("HR (CI), Ref. Grp. 1: ", round(HR.Ref1,digits=4), " (",

round(HRCIL.Ref1,digits=4), ", ", round(HRCIU.Ref1,digits=4), ")" )

print(DISP1)

DISP2<-paste("HR (CI), Ref. Grp. 2: ", round(1/HR.Ref1,digits=4), " (",

round(1/HRCIU.Ref1,digits=4), ", ", round(1/HRCIL.Ref1,digits=4), ")" )

print(DISP2)

rm(HR.Ref1, HRCIL.Ref1, HRCIU.Ref1, DISP1, DISP2)

}

#Example of function call

#Syntax: RevRef(HR with original ref, HR CI lower limit (orig.), HR CI upper limit (orig.))

#Example:

RevRef(1.47, 1.14, 1.90)

**#####################**

**#Method 1: Deriving CI from Reported HR and P-value**

GetCI <- function(HR, Pval) {

SELnHR <- log(HR)/qnorm((1-Pval/2), mean=0, sd=1)

HR.CIL <- exp(log(HR) + SELnHR*1.96)

HR.CIU <- exp(log(HR) - SELnHR*1.96)

DISP1 <- paste("HR (CI), p-value: ", round(HR,digits=4), " (", round(HR.CIL,digits=4),

", ", round(HR.CIU,digits=4), "), p = ", round(Pval,digits=4))

print(DISP1)

rm(HR, Pval, SELnHR, HR.CIL, HR.CIU, DISP1)

}

#Example of function call

#Syntax: GetCI(Hazard Ratio, Log-Rank p-value)

#Example:

GetCI(.68, 0.0032)

**#####################**

**#Method 2: Hazard Ratio as calculated using event counts**

M2EvCt <- function(Rr, Rc, Or, Oc, Pval) {

Dth.r <- Or / Rr

Dth.c <- Oc / Rc

Dth.Diff = Dth.r - Dth.c

Pmult <- ifelse(Dth.Diff < 0, -1, 1)

inv.Vr <- 1/(((Or + Oc)*Rr*Rc)/((Rr + Rc)^2))

O_E <- (sqrt((Or + Oc)*Rr*Rc)/(Rr + Rc)*(qnorm((1-Pval/2), mean=0, sd=1))*Pmult)

LnHR <- O_E*inv.Vr

HR <- exp(LnHR)

HR.CIL <- exp (LnHR - 1.96*sqrt(inv.Vr))

HR.CIU <- exp (LnHR + 1.96*sqrt(inv.Vr))

DISP1 <- paste("HR (CI), p-value: ", round(HR,digits=4), " (", round(HR.CIL,digits=4),

", ", round(HR.CIU,digits=4), "), p = ", round(Pval,digits=4))

print(DISP1)

rm(Rr, Rc, Or, Oc, Pval, Dth.r, Dth.c, Dth.Diff, Pmult, inv.Vr, O_E, LnHR, HR, HR.CIL, HR.CIU, DISP1)

}

#Example of function call

#Syntax: M2EvCt(Total # group 1 patients , Total # group 2 patients, # events group 1,

\# events group 2, Log-Rank p-value)

#Example:

M2EvCt(300, 300, 105, 133, 0.003)

**#####################**

**#Method 4a: Hazard Ratio as calculated using KM estimates**

M4aHR.KM <- function(Rr, Rc, Surv.KMr, Surv.KMc, Pval) {

Or <- (Rr - (Rr*Surv.KMr))

Oc <- (Rc - (Rc*Surv.KMc))

Dth.r <- Or / Rr

Dth.c <- Oc / Rc

Dth.Diff = Dth.r - Dth.c

Pmult <- ifelse(Dth.Diff < 0, -1, 1)

inv.Vr <- 1/(((Or + Oc)*Rr*Rc)/((Rr + Rc)^2))

O_E <- (sqrt((Or + Oc)*Rr*Rc)/(Rr + Rc)*(qnorm((1-Pval/2), mean=0, sd=1))*Pmult)

LnHR <- O_E*inv.Vr

HR <- exp(LnHR)

HR.CIL <- exp (LnHR - 1.96*sqrt(inv.Vr))

HR.CIU <- exp (LnHR + 1.96*sqrt(inv.Vr))

DISP1 <- paste("HR (CI), p-value: ", round(HR,digits=4), " (", round(HR.CIL,digits=4),

", ", round(HR.CIU,digits=4), "), p = ", round(Pval,digits=4))

print(DISP1)

rm(Rr, Rc, Or, Oc, Surv.KMr, Surv.KMc, Pval, Dth.r, Dth.c, Dth.Diff, Pmult,

inv.Vr, O_E, LnHR, HR, HR.CIL, HR.CIU, DISP1)

}

#Example of function call

#Syntax: M4aHR.KM(Total # group 1 patients , Total # group 2 patients, KM Survival group 1,

KM Survival group 2, Log-Rank p-value)

#Example:

M4aHR.KM(300, 300, 0.586, 0.445, 0.003)

**#####################**

**#Method 4b. Hazard Ratio calculated using median survival estimates**

M4bMedSurv <- function(Or, Oc, MSr, MSc) {

HR <- MSc/MSr

SELnHR <- sqrt((1/Or)+(1/Oc))

LnHR <- log(HR)

HR.CIL <- exp(LnHR - 1.96*SELnHR)

HR.CIU <- exp(LnHR + 1.96*SELnHR)

DISP1 <- paste("HR (CI): ", round(HR,digits=4), " (", round(HR.CIL,digits=4),

", ", round(HR.CIU,digits=4),")")

print(DISP1)

rm(Or, Oc, MSr, MSc, SELnHR, LnHR, HR, HR.CIL, HR.CIU, DISP1)

}

#Example of a function call

#Syntax: M4bMedSurv(# events group 1, # events group 2, Median survival for group 1, Median survival for group 2)

#Example:

M4bMedSurv(105, 133, 3.8, 2.5)

**Reviewer #3 Comment #2:** Finally, in method 1, it is noted that "For these reasons, we prioritized an adjusted HR using the largest sample that adequately addresses confounding (ie. whole patient population over a matched patient cohort when matching decreased the sample size)." This surprised me, as the whole point of matching is to reduce confounding in studies, even while reducing sample size. While the "whole patient population" will of course have a larger sample size, there is no guarantee that an analysis of such data will in fact bring the least biased results, and in fact failing to account for such variables (through matching or adjusting in a regression analysis) would likely lead to biased estimates of the true HR. I would therefore argue, as another reviewer has previously also argued, that priority for HR starts with adjusted or matched analyses, and only if no other data are available, then use the unadjusted HR estimates. The overall goal of a systematic review and possibly included meta-analysis is to obtain a best unbiased estimate of the treatment effect and therefore data extraction for such a SR/MA should also be clearly focused on that. The Cochrane Handbook also appears to prefer adjusted to unadjusted treatment effects, see e.g. [https://training.cochrane.org/handbook/current/chapter-06#section-6-3](https://urldefense.com/v3/__https:/training.cochrane.org/handbook/current/chapter-06*section-6-3__;Iw!!PEK3uBjM8x5RqC4b!VCUVNE2KXJkvHoN9Qf6qKNYaK5iw4_QkJEgmasCYh_xPZ5rN074C0HMgxAkXOTuU-Ec$)

Response to Reviewer #3 Comment #2: We agree that matched or adjusted HR should be used preferentially over an unadjusted HR. We apologize for the lack of clarity in our statement. Our comment was meant to address the case when a paper reported both an adequately adjusted HR and a matched HR for the same data set. To clarify, we have modified the statement in the “Method 1” section as follows:

"When multiple hazard ratios were reported, the statistical analysis that produced the hazard ratio was also captured (i.e., univariable, multivariable) to follow the extraction priority. It is important to determine an extraction preference a priori for when more than one hazard ratio is reported. Our criterion was to prioritize adjusted or matched analyses over unadjusted data, and when both adjusted and matched analyses were available, to maximize group size, because analyses using entire populations account for the relative frequency of case types, severity of disease, surgeon experience, etc. and the results are more generalizable. For these reasons, we prioritized an adjusted HR using the largest sample that adequately addresses confounding (ie. adjusted analysis using the whole patient population over a matched patient cohort when matching decreased the sample size)."

We also clarified that an adjusted or matched analysis is preferable to an unmatched analysis by modifying the Quality Control Methods section of the manuscript using wording similar to that of the reviewer, as follows:

“When more than one analysis was reported, we selected an adjusted or matched analysis preferentially, and only if no other data were available, unadjusted data; we chose the largest available analysis that adequately addressed cohort differences using the same hierarchy as listed above. When no adjustment or matching was performed, the comparability of the groups was determined by comparing baseline values for a list of covariates potentially related to oncologic outcomes.”

We also clarified this priority in supplemental appendix S4: Assumptions, Rules, and Tips as follows:

“Our Rule: Use an adjusted or matched HR over an unmatched HR. In instances where both an adjusted and a matched HR are provided, to maximize group size, use an adjusted HR using the whole patient population over a matched patient cohort when matching decreased the sample size.”

**Journal Requirements - References:** Please review your reference list to ensure that it is complete and correct. If you have cited papers that have been retracted, please include the rationale for doing so in the manuscript text, or remove these references and replace them with relevant current references. Any changes to the reference list should be mentioned in the rebuttal letter that accompanies your revised manuscript. If you need to cite a retracted article, indicate the article’s retracted status in the References list and also include a citation and full reference for the retraction notice.

Response to Journal Requirements: All references have been checked for completeness, and citations have been modified to add details as shown in the tracked changes version of the manuscript. There are no retracted references; however, the Parmar 1998 reference had an Erratum/correction published that corrected one of the equations that was not relevant for our work, and those details have been added to the end of the reference as follows:

“Parmar MKB, Torri V, Stewart L. Extracting summary statistics to perform meta‐analyses of the published literature for survival endpoints. Statistics in medicine. 1998;17(24):2815-34. Corrected: Stat Med. 2004 Jun 15;23(11):1817.”

We have confirmed that all references can be found by searching Pubmed or google using the citations as currently listed.

There was a glitch in our reference handling software; therefore, the following references have been corrected as follows:

Reference #2 has been corrected to:

“2.	Tewari A, Sooriakumaran P, Bloch DA, Seshadri-Kreaden U, Hebert AE, Wiklund P. Positive surgical margin and perioperative complication rates of primary surgical treatments for prostate cancer: a systematic review and meta-analysis comparing retropubic, laparoscopic, and robotic prostatectomy. Eur Urol. 2012;62(1):1-15.”

Reference #5 has been corrected to:

“5.	Williamson PR, Smith CT, Hutton JL, Marson AG. Aggregate data meta-analysis with time-to-event outcomes. Stat Med 2002;21:3337-51.”

The order of Ref #7 Parmar 1998 and Ref #8 Higgins Chapter 6 has been swapped.

Reference #12 for the Cochrane Handbook has been updated to the latest online version:

“Ref #12 Higgins JPT, Thomas J, Chandler J, Cumpston M, Li T, Page MJ, et al. Cochrane handbook for systematic reviews of interventions. 6.2 ed (updated February 2021): Cochrane, 2021. Available from [www.training.cochrane.org/handbook](http://www.training.cochrane.org/handbook).”

Ref #18 has been removed and references 19-24 have been re-numbered to references 18-23.

**Guidelines for resubmitting figure files:** While revising your submission, please upload your figure files to the Preflight Analysis and Conversion Engine (PACE) digital diagnostic tool, [https://pacev2.apexcovantage.com/](https://urldefense.com/v3/__https:/eur01.safelinks.protection.outlook.com/?url=https*3A*2F*2Fpacev2.apexcovantage.com*2F&data=04*7C01*7Capril.slee.15*40ucl.ac.uk*7C17102949ba8d4c59308d08d9bb34667e*7C1faf88fea9984c5b93c9210a11d9a5c2*7C0*7C0*7C637746656898887422*7CUnknown*7CTWFpbGZsb3d8eyJWIjoiMC4wLjAwMDAiLCJQIjoiV2luMzIiLCJBTiI6Ik1haWwiLCJXVCI6Mn0*3D*7C2000&sdata=3Au0dt477*2BT1Rw4KPqaLAm1DTy5pv4lESkEHEaXx2Ng*3D&reserved=0__;JSUlJSUlJSUlJSUlJSUlJSUl!!PEK3uBjM8x5RqC4b!VCUVNE2KXJkvHoN9Qf6qKNYaK5iw4_QkJEgmasCYh_xPZ5rN074C0HMgxAkXF2rg_2c$). PACE helps ensure that figures meet PLOS requirements. To use PACE, you must first register as a user. Registration is free. Then, login and navigate to the UPLOAD tab, where you will find detailed instructions on how to use the tool. If you encounter any issues or have any questions when using PACE, please email PLOS at [figures@plos.org](mailto:figures@plos.org). Please note that Supporting Information files do not need this step.

Response to Guidelines for resubmitting figure files: The three figures to be included in the main body of the manuscript have been run through the PACE tool. The PACE tool changed the resolution to 300 ppi, converted them to a valid TIF file, and changed the names to meet requirements.
